# Solitary plasmacytoma of the jaws: therapeutical considerations and prognosis based on a case reports systematic survey^☆^

**DOI:** 10.1016/j.bjorl.2018.05.002

**Published:** 2018-06-11

**Authors:** Eduardo Madruga Lombardo, Fábio Luiz Dal Moro Maito, Cláiton Heitz

**Affiliations:** a Pontifícia Universidade Católica do Rio Grande do Sul (PUCRS), Faculdade de Odontologia, Departamento de Cirurgia Bucomaxilofacial, Porto Alegre, RS, Brazil; b Pontifícia Universidade Católica do Rio Grande do Sul (PUCRS), Faculdade de Odontologia, Departamento de Patologia Oral, Porto Alegre, RS, Brazil

**Keywords:** Plasmacytoma, Plasma cell tumor, Multiple myeloma, Plasmocitoma, Tumor de células plasmáticas, Mieloma múltiplo

## Abstract

**Introduction:**

Solitary plasmacytoma is a rare malignant tumor of plasma cells with no evidence of systemic proliferation. There are two known subtypes: extramedullary solitary plasmacytoma and solitary bone plasmacytoma. The etiology is still unknown. Both lesions present a risk of progression to multiple myeloma. A number of approaches have been used for treatment of solitary plasmacytoma.

**Objective:**

To carry out a systematic review of the case reports described in the literature, focusing on therapeutic and prognostic aspects.

**Methods:**

A search of clinical case reports was performed in the PubMed database using *Mesh Terms* related to “plasmacytoma” under the following criteria: type of study (case report), articles in English language, conducted in humans, with no publication date limits.

**Results:**

Of the 216 articles found, only 21 articles met the pre-established inclusion criteria.

**Conclusion:**

The occurrence of solitary bone plasmacytoma in the bones of the face is a rare condition prevalent between the 4th and 6th decades of life, located in the posterior region of the mandible in most cases. Histopathological examination and systemic investigation are mandatory for confirmation of diagnosis.

## Resumo

**Introdução:**

O plasmocitoma solitário é um tumor maligno raro de células plasmáticas sem evidência de proliferação sistêmica e engloba dois subtipos: plasmocitoma solitário extramedular e plasmocitoma solitário ósseo. A etiologia ainda é desconhecida. Ambas as lesões apresentam risco de progressão para mieloma múltiplo. Uma série de abordagens tem sido usada para seu tratamento.

**Objetivo:**

Realizar uma revisão sistemática da literatura com enfoque nos aspectos terapêuticos e prognósticos.

**Método:**

Realizou-se uma busca de relatos de caso clínico na base de dados PubMed com termos de busca relacionados com “plasmocitoma” sob os seguintes critérios: tipo de estudo (relato de caso), artigos na língua inglesa, estudos realizados apenas em humanos, sem limites de data de publicação.

**Resultados:**

Dos 216 artigos encontrados, apenas 21 artigos preencheram os critérios de inclusão pré-estabelecidos.

**Conclusão:**

A ocorrência de plasmocitoma solitário ósseo nos ossos da face é uma condição rara prevalente entre a 4^a^ e a 6^a^ décadas de vida, localizada na região posterior de mandíbula na maioria dos casos. O exame histopatológico e a investigação sistêmica são mandatórios para confirmação do diagnóstico.

## Introduction

Solitary plasmacytoma (SP) is a rare malignant tumor of plasma cells with no evidence of systemic proliferation. When there is systemic involvement, that is, the involvement of multiple skeletal sites, the disease is called multiple myeloma (MM), one of the most frequent presentations of neoplasia of the plasma cells.^1^

The SP presents an incidence of 2–5% of all neoplasms and two subtypes: extramedullary solitary plasmacytoma (ESP) and solitary bone plasmacytoma (SBP).^1^, ^2^ ESP originates from soft tissues and is more frequent in the head and neck region, specifically in the upper respiratory tract, whereas the SBP presents as an intramedullary bone lesion in the axial skeleton or pelvic bones.^3^

The etiology of solitary plasmacytoma is unknown, however, it is suggested that chronic stimulation, radiation overdose, viral infections and genetic interaction in the reticuloendothelial system may contribute to the development of the lesion.^4^

The SBP has a predilection for males between the 6th and the 7th decades of life, however, it can affect individuals of any age. Patients affected by SBP, in general, present a primary complaint of swelling associated with minimal pain.^5^, ^6^

The SBP can present two radiographic patterns: the first can be a delimited radiolucent area; the second, as a destructive lytic mass in the mandible. Microscopically, monoclonal proliferation of plasmacytoid cells with eccentric nuclei and basophilic cytoplasm are observed.^7^, ^8^

Once the biopsy is performed and the histopathological diagnosis of SBP is defined, it is important to submit the patient to systemic investigation of disseminated disease through imaging examinations of the whole body, bone marrow biopsy, complete hematological examination and electrophoresis of urine and plasma to screen proteins synthesized by tumor cells.^1^, ^9^, ^10^, ^11^

Although MM is a relatively common occurrence when compared to other plasma cell neoplasms, SBP in the skull bones is a relatively rare entity with very little published literature.^12^ The objective of the present study is to perform a systematic review of case reports focusing on its epidemiology, clinical and microscopic characteristics, as well as its diagnosis, treatment, prognosis and the importance of longitudinal clinical follow-up.

## Methods

A systematic review of the case reports was performed from articles found in the PubMed/MEDLINE database. A search strategy was developed using the Pubmed Advanced Search Builder with the following combinations of the Mesh terms “Plasmacytoma”, “Myeloma”, “mandible” and “maxilla” and the derived entry terms in conjunction with the Boolean operators “OR” And “AND”, as described in Table 1. The inclusion criteria applied for the case reports were: type of study (case reports), in English language and conducted in humans.

**Table 1:** Search strategy on Pubmed Advanced Search Builder.

Filter “All fields”	Mesh Term	Entry terms
1	Plasmacytoma	Plasmacytomas OR Plasmocytoma OR Plasmocytomas OR Plasma Cell Tumor OR Plasma Cell Tumors OR Tumor, Plasma Cell OR Tumors, Plasma Cell
**Boolean operator “OR”**		
2	Myeloma	Multiple Myelomas OR Myelomas, Multiple OR Myeloma, Multiple OR Myeloma, Plasma-Cell OR Myeloma, Plasma Cell OR Myelomas, Plasma-Cell OR Plasma- Cell Myeloma OR Plasma-Cell Myelomas OR Myelomatosis OR Myelomatoses OR Plasma Cell Myeloma OR Cell Myeloma, Plasma OR Cell Myelomas, Plasma OR Myelomas, Plasma Cell OR Plasma Cell Myelomas OR Kahler Disease OR Disease, Kahler OR Myeloma-Multiple OR Myeloma Multiple OR Myeloma-Multiples
**Boolean operator “AND”**		
3	Mandible	Mandibles OR Mylohyoid Ridge OR Mylohyoid Ridges OR Ridge, Mylohyoid OR Ridges, Mylohyoid OR Mylohyoid Groove OR Groove, Mylohyoid OR Grooves, Mylohyoid OR Mylohyoid Grooves OR mandible OR Lower jaw
**Boolean operator “OR”**		
4	Maxilla	Maxillas OR Maxillary Bone OR Bone, Maxillary OR Bones, Maxillary OR Maxillary Bones OR Maxillae OR Upper Jaw OR Maxilla
**Boolean operator “AND”**		
5	–	Solitary

## Results

Based on the search strategy, 216 articles were found. Applying the inclusion criteria as filter, 114 articles were excluded. The remaining 102 articles were submitted to selective reading of the titles, which determined the exclusion of 78 articles. The remaining 24 articles were submitted to analysis of the abstracts and, at this stage, three articles were excluded because they presented SP lesion located in an anatomical region distinct from the oral and maxillofacial surgeons’ expertise.

The interpretative reading of the full case reports was carried out in 21 articles: 20 case reports and 1 case series. The case reports were arranged in descending order of the year of publication in two tables: one providing epidemiological and clinical data as well as the initial diagnosis (Table 2); the other (Table 3) containing information regarding the detection of M-protein (or paraprotein), therapeutic approach, follow-up time, recurrences and evolution for MM, as well as findings considered relevant to each article. In addition, the case series was included in the discussion of this same study.

**Table 2:** Epidemiological and clinical data.

Reference	Age (years)	Sex	Race	Localization	Clinical aspects	Evolution time	Imaging aspects	Initial diagnosis
Cioranu et al.^13^ (2013)	52	M	NR	Zygomatic, molar and orbital region, right side	Swelling	2 years	Expansive mass	Plasmacytoma
S An et al.^14^ (2013)	65	F	NR	Angle, ramus and coronoid process of the mandible, left side	Swelling	2 years	Poorly defined radiolucent lesion	NR
Nanda et al.^15^ (2012)	70	F	NR	From canine to molar with palatine involvement, right side	Swelling	15 days	Misty radiolucent lesion	Abscess
Pinto et al.^16^ (2007)	65	F	Black	Superior canine region, left side	Swelling and pain	15 days	Diffuse radiolucent lesion	NR
Poggio et al.^17^ (2007)	75	F	NR	Anterior border of the mandible, right side	Swelling and pain	3 years	Radiolucent lesion related to an implant	NR
Anil^2^ (2007)	52	M	NR	Superior premolar and molar region extending to the palate, right side	Swelling and pain	NR	Diffuse radiolucent lesion	NR
Canger et al.^18^ (2007)	76	F	NR	Anterior region of the mandible	Swelling, pain and erythema	6 months	Multilocular radiolucent lesion	NR
Ozdemir et al.^4^ (2005)	63	F	NR	Palate	Swelling	NR	Lytic bone lesions	NR
Yoon et al.^19^ (2003)	15	M	Asian	Inferior molar region, right side	Swelling	6 years	Increase in periodontal ligament space between lower molars	Granuloma pyogenic
Matsumura et al.^20^ (2000)	83	M	NR	Maxillary sinus, right side	Swelling	1 month	Velvet of the right maxillary sinus and aspect of “honeycombs”	Myxoma or ameloblastoma
Ho et al.^21^ (1999)	22	M	Asian	Mandibular ramus, right side	Swelling	6 months	Osteolytic lesion	NR
Millesi et al.^22^ (1997)	44	M	NR	Premolar region to the angle in mandible, left side	Swelling	NR	Osteolytic lesion	NR
Kanazawa et al.^23^ (1993)	49	F NR	From medium line to the ramus	of the mandible, left side	Swelling 2 years	Expansive radiolucent lesion with	“Soap bubbles” aspect	Myxoma
Saito et al.^24^ (1987)	52	F	NR	From premolar region to maxillary tuberosity, left side	Swelling	4 years	Osteolytic multilocular lesion	NR
Mustoe et al.^25^ (1984)	47	F	Black	Maxillary sinus, left side	Increase of volume, pain and headache	NR	Presence of radiopaque sclerotic mass within the maxillary sinus	Orbital pseudotumor
Loh^26^ (1984)	36	M	Asian	Lower central incisors region	Swelling	1 year	Radiolucent lesion with defined margins cropped out	Ameloblastoma
Christensen et al.^27^ (1987)	34	M	NR	Body of the mandible, left side	Swelling	1 year	NR	NR
Raley and Granite ^28^(1977)	34	M	Black	Maxillary tuberosity, right side	Swelling	1 year	Trabecular pattern changed	NR
Lipper et al.^29^ (1975)	64	M	White	Mandible, left side	Swelling	6 months	Radiopaque mass	Osteosarcoma
Webb et al.^30^ (1966)	Case 1: 59	Case1: F	NR	Case 1: ramus and angle of the mandible, right side	Case 1: pressure sensation	Case 1: 10 months	Case 1: tumoral mass	NR
	Case 2: 56	Case 2: F		Case 2: ramus and angle of the mandible, right side	Case 2: Swelling	Case 2: 9 months	Case 2: multilocular lesion

**Table 3:** Detection of M-protein (or paraprotein), therapeutic approach, follow-up time, recurrences and evolution for MM, as well as findings considered relevant to each article.

Reference	Presence of M-protein	Treatment	Follow up	Recurrences	Evolution to MM	Relevant findings
Cioranu et al.^13^ (2013)	NR	Chemotherapy, Surgical excision and autotransplantation	2 years/patient deceased	No	Previously diagnosed with MM	Recurrent lesions in other bones; patient accompanied by hematologist for 14 years
An et al.^14^ (2013)	Positive	Chemotherapy	8 months	No	Diagnosed with MM through a lesion in the mandible	Solitary lesion with systemic signs of MM
Nanda et al.^15^ (2012)	Negative	Partial maxilectomy	1 year	No	No	Diagnosis of extramedullary plasmacytoma
Pinto et al.^16^ (2007)	Positive (blood)/Negative (urine)	Chemotherapy	9 months/patient deceased	No	Yes	Diagnosis of MM with plasmacytoma
Poggio et al.^17^ (2007)	Negative	Radiotherapy	6 months	No	No	Patient with a history of bone plasmocytoma in the spine (12 years ago)
Anil^2^ (2007)	Negative	NR	5 years	No	No	–
Canger et al.^18^ (2007)	Negative	Patient deceased before treatment started	6 months/patient deceased	No	No	Patient submitted to previous surgical excision of plasmacytoma located in the iliac
Ozdemir et al.^4^ (2005)	Negative	Chemotherapy	NR	NR	No	–
Yoon et al.^19^ (2003)	Negative	Dose reduction of immunosuppressants and radiotherapy	7 years	No	No	Patient underwent through renal
Matsumura et al.^20^ (2000)	Positive	Radiotherapy e chemotherapy	12 months	No	No	The lesion decreased but did not disappear
Ho et al.^21^ (1999)	Positive	Radiotherapy e chemotherapy	28 days	No	Diagnosed with MM through a lesion in the mandible	The lesion decreased but did not disappear
Millesi et al.^22^ (1997)	Negative	Radiotherapy e chemotherapy e surgical resection	4 years	No	No	Patient underwent through reconstruction and oral rehabilitation
Kanazawa et al.^23^ (1993)	Positive	Radiotherapy and hemimandibulectomy	NR	No	No	Patient underwent
Saito et al.^24^ (1987)	Positive	Excisional biopsy with 1 cm margin	3 years and 6 months	Yes (1 month after surgery)	No	Recurrence of the lesion was treated with effective radiotherapy
Mustoe et al.^25^ (1984)	Negative	Radiotherapy	NR	No	No	–
Loh^26^ (1984)	Negative	Surgical excision and radiotherapy	3 years	No	No	–
Christensen et al.^27^ (1987)	NR	Surgical curettage	3 years	Yes	No	The case report is focused on the recurrent lesion. The treatment was radiotherapy.
Raley and Granite^28^ (1977)	Positive	Surgical excision and radiotherapy	NR	No	No	–
Lipper et al.^29^ (1975)	Positive	Hemimandibulectomy	9 months	No	No	–
Webb et al.^30^ (1966)	Case 1: Negative	Case 1: Hemimandibulectomy	Case 1: 18 months/patient deceased	Case 1: No	Case 1: Yes	–
	Case 2: Negative	Case 2: Surgical curettage and radiotherapy	Case 2: 9 months	Case 2: No	Case 2: No	

## Discussion

### Epidemiological data

The distribution of SBP cases by age ranged from 15 to 83 years, with a mean age of 54.15 years for both sexes, 43.6 years for men and 61.54 years for women. The highest incidence of SBP occurred between the 4th and 6th decades of life.^2^, ^4^, ^13^, ^14^, ^15^, ^16^, ^17^, ^18^, ^19^, ^20^, ^21^, ^22^, ^23^, ^24^, ^25^, ^26^, ^27^, ^28^, ^29^, ^30^ The distribution by gender was balanced, accounting for 11 cases in women and 10 in men. These data corroborate the results obtained by Dores et al.^12^

The patient's race was reported in only 7 case reports: 3 black patients, 3 Asian patients and 1 white patient.^2^, ^4^, ^13^, ^14^, ^15^, ^16^, ^17^, ^18^, ^19^, ^20^, ^21^, ^22^, ^23^, ^24^, ^25^, ^26^, ^27^, ^28^, ^29^, ^30^ Although most of the case reports did not report the race of the patients, a predilection of the SBP for white individuals is observed in the literature.^12^

### Location of the lesion

In the case reports reviewed, the most common site of SBP appearance was the mandible, more precisely in posterior regions.^2^, ^4^, ^13^, ^14^, ^15^, ^16^, ^17^, ^18^, ^19^, ^20^, ^21^, ^22^, ^23^, ^24^, ^25^, ^26^, ^27^, ^28^, ^29^, ^30^ These findings confirm those found by Loh^26^ and Pisano et al.^31^

Although the literature presents pain as the main symptom.^2^ The present study found painless increase in volume as the most common clinical finding. Headache and pressure sensation have also been reported.^25^, ^30^ The time of evolution of the lesion ranged from 15 days to 72 months, with an average time of evolution of 15.11 months.^2^, ^4^, ^13^, ^14^, ^15^, ^16^, ^17^, ^18^, ^19^, ^20^, ^21^, ^22^, ^23^, ^24^, ^25^, ^26^, ^27^, ^28^, ^29^, ^30^, ^31^

### Imaging aspects and initial diagnosis

Radiographically, it was observed that, in most studies, the solitary plasmacytoma appears as a diffuse, multilocular radiolucent lesion. Bone destruction seems to be limited to the medullary region of the skull bones.^2^, ^4^, ^13^, ^14^, ^15^, ^16^, ^17^, ^18^, ^19^, ^20^, ^21^, ^22^, ^23^, ^24^, ^25^, ^26^, ^27^, ^28^, ^29^, ^30^

Only 8 case reports presented a presumptive clinical diagnosis, and the hypotheses presented were: abscess, pyogenic granuloma, myxoma, ameloblastoma, orbital pseudotumor and osteosarcoma.^13^, ^15^, ^19^, ^20^, ^23^, ^25^, ^26^, ^29^

Differential diagnosis of PBS should be performed in relation to other lesions that are similar in the routine imaging exams such as ameloblastoma, keratocystic odontogenic tumor, myxoma, giant cell central lesion, metastatic tumors, vascular malformation, sarcoma and lymphoma.^32^, ^33^ Thus, the histopathological examination becomes essential for the definitive diagnosis.

### Presence of M-protein

Plasma protein M or paraprotein, monoclonal immunoglobulin synthesized by tumor cells, was investigated in 18 cases.^2^, ^4^, ^13^, ^14^, ^15^, ^16^, ^17^, ^18^, ^19^, ^20^, ^21^, ^22^, ^23^, ^24^, ^25^, ^26^, ^27^, ^28^, ^29^, ^30^ The presence of the M-protein was reported in 8 case reports,^14^, ^16^, ^20^, ^21^, ^23^, ^24^, ^28^, ^29^ corresponding to 38.09%. This rate is in the range of 24–72% indicated in other studies.^5^

The presence of M-protein is obtained by examination of electrophoresis from blood or urine samples.^33^ The use of this exam to determine the diagnosis of SBP is still inexact since the presence of paraprotein does not always determine the existence of the disease in question, however, it should be emphasized that its diagnostic value is relevant in cases where it is desired to evaluate the presence of M-protein.^1^, ^34^

There are authors who advocate that the presence of paraprotein even after treatment may be indicative of residual tumor or hidden.^1^, ^35^

### Treatment

The treatment used for the cases were the following:

• Only radiotherapy – 2 cases.^17^, ^25^
• Only chemotherapy – 3 cases.^4^, ^14^, ^16^
• Only surgical intervention – 5 cases.^5^, ^24^, ^27^, ^29^, ^30^
• Surgical intervention associated to radiotherapy – 4 cases.^23^, ^26^, ^28^, ^30^
• Radiotherapy associated to chemotherapy – 2 cases.^20^, ^21^
• Surgical intervention, radiotherapy and autotransplantation – 1 case.^13^
• Radiotherapy and decreased dosage of immunosuppressors – 1 case.^19^
• Radiotherapy, chemotherapy and surgical intervention – 1 case.^22^

In one of the case reports the therapeutic approach was not reported^2^ and in an other, the patient deceased before starting treatment.^18^ The ideal therapeutic approach is still controversial, however, radiotherapy seems to be the treatment that offers better clinical results since the SBP reveals itself as a radiosensitive lesion.^5^, ^36^ The rates of local control of SBP with radiotherapy presented in the literature exceed the range of 80%.^2^, ^37^, ^38^ Surgical intervention should be carried out in situations where there is no prediction of functional or esthetic damage.^23^

Chemotherapy is advocated only on the basis of reports in the literature that showed improvement of local control and delayed development of MM.^38^ However, chemotherapy alone has no benefit compared to radiotherapy but when instituted adjunctively it appears to offer beneficial effect in patients with a higher risk of treatment failure, that is, those with tumor lesions greater than 4–5 cm.^1^, ^38^, ^39^

### Prognosis and follow-up

Follow-up time after treatment ranged from 28 days to 7 years. The mean follow-up period was 19.9 months. There were 4 deaths.^13^, ^16^, ^18^, ^19^

In only 2 cases (9.5%) did SBP evolve to MM.^16^, ^30^ The low incidence of progression to MM in skull bones damage was reported in the same way in other retrospective studies.^23^, ^26^

Frequently, SBP can be found as a radiographic finding and can represent a primary lesion or focus of MM as previously reported.^40^, ^41^ The present study revealed that in 2 case reports, MM was diagnosed from the detection of a skull bone lesion.^14^, ^21^

There were 2 reports of recurrence of the lesion, and in one case the event occurred in 1 month and in another after 3 years, both treated surgically.^24^, ^27^ The mean time to recurrence of lesion after treatment reported in other studies was 2–2.5 years.^42^

The worst prognosis corresponds to progression from SBP to MM. Such event is directly related to the size of the tumors. Scientific evidence suggests that patients who present tumor masses, previously diagnosed as SBP, with a size larger than 4–5 cm have a higher risk of developing MM.^39^ In addition, the bone location of the plasmacytoma in comparison with the extramedullary entity, age (patients over 60 years) and the presence of paraprotein at the time of diagnosis also determine higher progression rates for MM.^36^, ^43^

## Final considerations

SBP is a rare condition in the bones of the face. It affects patients between the 4th and 6th decades of life without predilection for gender. The lesion arises mainly in the mandible, more precisely in the posterior region. Commonly, it presents as a multilocular radiolucent lesion. The main sign associated with the development of SBP is painless volume increase. Biopsy and histopathologic examination are mandatory since the definition of diagnosis determines the need for advanced investigation to rule out the possibility of MM.

The importance of early diagnosis is justified in that the plasmacytoma may be a primary or metastatic lesion of MM. The treatment of choice for SBP is radiotherapy. The association of surgical intervention and chemotherapy is reserved for specific cases. Periodic follow-up of the patient is necessary for at least 3 years after diagnosis due to the possibility of developing MM.

## Conflicts of interest

The authors declare no conflicts of interest.
